# *Trachyspermum ammi* aromatic water: A traditional drink with considerable anti-*Candida* activity

**DOI:** 10.18502/cmm.6.3.3979

**Published:** 2020-09

**Authors:** Ali Arabi Monfared, Seyyed Amin Ayatollahi Mousavi, Kamiar Zomorodian, Davood Mehrabani, Aida Iraji, Mahmood Reza Moein

**Affiliations:** 1 Department of Medical Mycology and Parasitology, School of Medicine, Kerman University of Medical Sciences, Kerman, Iran; 2 Medical Mycology and Bacteriology Research Center, Kerman University of Medical Sciences, Kerman, Iran; 3 Basic Sciences in Infectious Diseases Research Center, School of Medicine, Shiraz University of Medical Sciences, Shiraz, Iran; 4 Stem Cell and Transgenic Technology Research Center, Shiraz University of Medical Sciences, Shiraz, Iran; 5 Department of Pharmacognosy, Shiraz University of Medical Sciences, Shiraz, Iran

**Keywords:** Antifungal activity, Aromatic water, Biofilm, *Candida*, Candidiasis, *Trachyspermum ammi*

## Abstract

**Background and Purpose:**

Aromatic waters (AWs) are therapeutic distillates, which harbor both essential oil and water-soluble components of a plant. Due to the dispersion of the light amount of essence through the AWs, they have their specific pleasant smell, taste, and medicinal properties. In Iranian traditional medicine, *Trachyspermum ammi* AW is used to treat gastrointestinal disorders. The present study was conducted to determine the chemical composition of the essential oil extracted from *T. ammi* AW and its antifungal activities against *Candida* species.

**Materials and Methods:**

The composition of the essential oil extracted from *T. ammi* AW was analyzed by gas chromatography-mass spectrometry. In addition, the evaluation of the antifungal activity of AW against *Candida* species was performed using broth microdilution methods as recommended by the Clinical Laboratory Standard Institute. Moreover, the biofilm formation inhibition, antioxidant properties, and experimental activity of AW were determined in an animal model.

**Results:**

According to the results, thymol (78.08%) was the major compound of EO, followed by carvacrol (8.20%) and carvotanacetone (6.50%). Furthermore, *T. ammi* AW exhibited antifungal activities against the examined fungi and inhibited the biofilm formation of C. albicans at a concentration of up to 0.25 V/V. Histopathological analyses revealed that *Candida* colonization declined in the mice following the administration of*T. ammi* AW in a therapeutic trial.

**Conclusion:**

It seems that the presence of phenolic monoterpenes in AW has resulted in antifungal effects. Pleasant odor and antioxidant properties are extra bonuses to the antimicrobial effects of this plant. Based on the findings, AW might have the potential to be used in the management of alimentary candidiasis or oral hygienic products.

## Introduction

Ifections caused by opportunistic yeasts in particular candidiasis are among the most common human fungal infections. The infection, which is usually caused by endogenous mucosal *Candida* species, especially *C. albicans* [ 1 ], includes a broad spectrum of clinical manifestations ranging from simple dermatosis to life- threatening candidemia, with a relatively high mortality rate [ 2 , 3 ]. Oral and esophageal candidiasis are the most frequent forms of this infection. Gastrointestinal candidiasis is another form of mucocutaneous candidiasis. This form of candidiasis encompasses a range of clinical symptoms, epigastric, abdominal pain, nausea, vomiting, bloating, and intestinal cramps. Moreover, some cases have such symptoms as rectal itch, fever, chills, and abdominal mass, occurring in individuals with various underlying risk factors involving the gastrointestinal (GI) tract [ 4 , 5 ].

Gastritis caused by *Candida* species colonization is another phenomenon that happens in the acidic pH of the gut of individuals taking anti-acid drugs, such as H2 blockers. These commensal yeasts residing in the GI tract have been reported to run away from their niche to the bloodstream and cause invasive candidiasis. In this regard, Miranda et al. reported identical strains of *Candida* isolated from both the blood and rectal culture of patients [ 6 ]. Moreover, it has been estimated that about one-third of patients with fulminant hepatic failure may develop fungal infections, with *C. albicans* being the most frequent agent [ 7 ]. The treatments of choice for GI candidiasis are azole and polyene antifungals. However, as a result of the recent emergence of resistance to these routine antifungal drugs among *Candida* species [ 8 ], the management and treatment of this infection are still considered a universal crisis. A strategy to overcome this problem is the use of novel antimicrobial agents, especially from natural resources.

Middle Eastern countries, including Iran, have unique botanical flora, used as remedies and syrups in Iranian traditional medicine for thousands of years. It has been shown previously that many of these medicinal plants and their aromatic products have antimicrobial properties. In addition, essential oils (EOs) distilled from these aromatic plants have been used in medicine and aromatherapy, as well as in cosmetics and food industries. These EOs are rich in terpenoid compounds, in particular monoterpenes, with known antimicrobial properties [ 9 , 10 ]. Aromatic water (AW) harboring a slight amount of EO is the therapeutic distillates of aromatic plants. Not only has been AW popularly used for medicinal purposes for decades but also the sweetened form of this AW distilled from endemic fragrant plants is used as a pleasant beverage by Iranians, especially on hot summer days.

*Trachyspermum ammi* is a grassy annual plant of Umbelliferae family with a white flower and small brownish seeds, which commonly grows in Iran, India, Egypt, and Europe [ 11 ]. The seeds are used for their flavor and spice in the food industry [ 11 ]. In Persian traditional medicine, the seeds of *C. copticum* ('Zenyan' in Persian) were used for their therapeutic effects, such as diuretic, anti-vomit, carminative, anthelmintic, expectorant, analgesic, anti-asthma, anti-dyspnea, and anti-spasm impacts [ 12 ]. Moreover, this plant is used for the treatment of diarrhea, colic, and other bowel problems [ 12 , 13 ].

The EO of *C. copticum* has been reported to have antioxidant [ 14 ], anti-cholinergic [ 15 ], anti-histaminic [ 16 ], and analgesic effects [ 17 ]. In addition, the EO distilled from *C. copticum* successfully inhibited the growth of bacteria associated with gastrointestinal infections, including *Staphylococcus aureus*, Entero- pathogenic *Escherichia coli, Salmonella typhimurium* [ 18 ], multi-drug resistant S. typhi [ 19 ], and Helicobacter pylori [ 20 ]. It has been also shown that *C. copticum* EO has inhibitory effects against fungal growth [ 21 , 22 ] and aflatoxin production by *Aspergillus parasiticus* [ 23 ]. Furthermore, *C. copticum* seeds demonstrate anthelmintic activity against human *Ascaris lumbricoides* and gastrointestinal nematodes of sheep [ 24 , 25 ].

In previous studies [ 21 - 23 ], the EO distilled from *T. ammi* revealed considerable antifungal activities. Regarding such activity and a global tendency toward using natural products and phytochemicals, the present study was conducted to evaluate the chemical composition, as well as the antioxidant and antifungal activities of *T. ammi* AW against *Candida* species. Moreover, the in vivo efficacy of AW in the prevention and treatment of GI candidiasis was assessed experimentally in the mice infected with *C. albicans*.

## Materials and Methods

**Plant material**

The plant used in this study was collected from Meymand, Fars providence, Iran, and identified and confirmed by an expert botanist. A voucher specimen was recorded in the herbarium (Voucher no. HSUMS 301).

**Aromatic water extraction**

The aerial parts of the plant (10 kg of plant material with 30 liters of water) were hydrodistillated, by means of an industrial apparatus from the Nab Factory (Meymand, Iran). This method is an old and feasible technique for the distillation of EO and preparation of edible AW in Iran and some other countries. Briefly, plant material was submerged in water, and the mixture was heated for 6 h to produce steam carrying the most volatile chemicals. The steam was then cooled down to collect the AW containing distilled compounds.

**Extraction of essential oil from aromatic water and analysis**

To extract EO from AW, 300 ml of the AW was transferred into a decanter, and active compounds were extracted by a solvent (300 ml of diethyl ether, 3 times). The solvent was removed by an evaporator. Finally, EO was dried over anhydrous sodium sulfate. The analysis of EO was performed using an Agilent gas chromatograph series 7890A coupled with 7000 Triple Quad mass spectrometer. A fused silica capillary DB- 1MS column (30 m, 0.25-mm inside diameter; 0.25 µm film thickness) was used for the separation of the different compounds of EO. The injector and auxiliary temperature were kept at 250℃ and 280℃, respectively. Helium was used as the carrier gas at a flow rate of 1.2 ml/min. The oven temperature was programmed to increase from 60℃ to 280℃ at a rate of 4℃/min and kept at this temperature for 4 min. The split ratio was 1:30, and the mass spectra were recorded over a range of 46-650 amu, with an ionizing voltage of 70 eV.

The compounds of EO were identified by comparing retention indices with those reported in the literature, mass spectra with the Wiley library, and published mass spectra data. The retention indices were determined using the retention times of n-alkanes that were injected after EO administration under the same chromatographic conditions. The retention indices for all components were determined according to the Van Den Dool method using n-alkanes (C8 to C26) as standard. Relative percentage amounts were calculated from the total area under the peaks by the apparatus software.

**Determination of antifungal activity**

The antifungal activities of AW were determined against 16 standard strains of *Candida, including C. ablicans, C. tropicalis, C. krusei, C. glabrata, C. dubliniensis, and C. parapsilosis*. In addition, the antifungal activities of AW were tested against six clinical isolates of yeasts identified by sequencing method. Additionally, fluconazole was used as a positive control.

**Determination of minimum inhibitory concentration**

The MICs of AW against the standard and clinical species of *Candida* were determined by the broth micro dilution method as recommended by the clinical and laboratory standards institute, with some modifications [ 26 ]. The serial dilutions of AW (1/2 to 1/1024 V/V) were prepared in 96-well microtiter trays using RPMI- 1640 (Sigma, St. Louis, USA) buffered with 3- morpholinopropane-1-sulfonic acid (MOPS) (Sigma, St. Louis, USA). The growth in each well was compared with that of the control well. The MICs were visually determined and defined as the lowest concentration of AW that produced no visible growth. Each experiment was performed in triplicate. In addition, the minimum fungicidal concentration (MFC) of the examined AW was determined by culturing 10 µL from the wells, showing no visible growth, onto Sabouraud dextrose agar (SDA) plates. The MFCs were determined as the lowest concentration yielding no more than four colonies, which yielded a yeast mortality of about 98% in the initial inoculums.

**Inhibition of biofilm formation**

Following a study performed by Ramage et al. [ 27 ], *C. albicans* (CBS1905) was cultured and maintained on SDA (Merck, Germany) plate. After 48 h, one loop of colonies was transferred to 20 mL Sabouraud dextrose broth (Merck, Germany) and incubated overnight in an orbital shaker incubator (Jal-Tajhiz, Iran) (100 rpm) at 30ºC under aerobic condition. Yeast cells were harvested and washed twice in sterile phosphate- buffered saline (PBS). Then, they were resuspended in RPMI 1640 supplemented with L-glutamine (Gibco) and buffered with MOPS. Subsequently, the cell densities were adjusted to 1.0×106 cells/ml after counting with a hematocytometer. Serial dilution of AW (1/2 - 1/1024 V/V) in RPMI 1640 was prepared in presterilized, polystyrene, flat-bottom, 96-well microtiter plates (SPL Life Sciences, Korea). After the addition of 0.1 ml of the yeast inoculums to the wells, the tray was incubated at 30ºC for 24-48 h in a humid atmosphere. In the next stage, 200 μl of the un- inoculated medium was included as a negative control (blank). In addition, RPMI with yeasts but without AW was served as the positive control.

**Biofilm inhibition assay**

A semi-quantitative measure of biofilm formation was assayed using a 2, 3-bis (2-methoxy-4-nitro-5- sulfophenyl)-2H-tetrazolium-5-carbox-anilide (XTT) reduction assay. The XTT (Sigma, USA) was prepared as a saturated solution at a concentration of 0.5 mg/mL in Ringer's lactate. Prior to each assay, an aliquot of XTT stock solution was thawed and treated with menadione sodium bisulfate (10 mM prepared in Distilled Water; Sigma, USA) to obtain a final concentration of 1 µM menadione. A 100-µL aliquot of XTT-menadione was then added to each pre-washed well. Subsequently, the plates were incubated in dark for 2 h at 37°C, and the colorimetric change at 490 nm (a reflection of the metabolic activity of the biofilm) was measured with a microplate reader (POLAR star Omega, BMG Labtech, Germany) [ 28 ].

**Antioxidant assays**

The free radical-scavenging activity of EPs was determined by the DPPH assay as described previously [ 28 , 29 ]. Briefly, 270 µL of the different dilutions of EO was mixed with a methanolic solution of DPPH (100 µM) and incubated at room temperature for 30 min. The absorbance of the samples was measured at 517 nm by a spectrophotometric method. The IC50 values were calculated by the Curve-Expert software (for Windows, version 1.34). In addition, the AW percentage of inhibition was determined.

**Animal modeling**

In this study, 27 female mice (BALB/c) with the age of 4 weeks and an approximate weight of 19-23 g were obtained from the Laboratory Animal Center of Shiraz University of Medical Sciences, Shiraz, Iran. The mice were kept at 25-30ºC and fed normal diet. To investigate the effect of *T. ammi* AW on alimentary candidiasis, 27 infected mice were randomly allocated into three treatment groups at separate cages, including test group (n=9) that received T. ammi AW instead of drinking water, positive control group (n=9) administered with 1 g/ml fluconazole in drinking water, and negative control group (n=9) that remained untreated and received only drinking water. As the healthy mice were resistant to colonization by yeast (*C. albicans*), antibiotic and immunosuppressive drugs were administrated prior to inoculation.

For this purpose, 3 days before the insemination of the yeast cell suspension, the mice were treated with an antibiotic (1 g/L tetracycline, 0.1 g/L gentamicin, and 2 g/L streptomycin). In the next step, 100 ml of yeast cell suspension at a concentration of 2×108 cells was intragastrically inoculated into the stomach of each mouse by the gavage feeding needle. Immediately following yeast gavage, the mice were injected with cyclophosphamide (100 mg/kg) intraperitoneally. The injections were repeated 1 week prior to sacrifice [ 30 ]. Three mice from each group were euthanized by injectable anesthetic overdose (3x anesthesia dose; 80-100/10 mg/kg) of ketamine /xylazine (intraperitoneally) at the end of the 3rd, 4th, and 5th weeks of fungal challenges. The stomach was removed, and a part of the stomach specimen was placed in formalin for histological studies. The other part was weighed under sterile conditions and homogenized in the tube containing sterile PBS (500 mg/ml) with a homogenizer (SpeedMill plus-Analytik Jena, Germany). The homogenized specimens were immediately cultured onto SDA (Merck, Germany) plates. Following 24-48h of incubation at 32°C, CFUs/500 mg was determined. The number of colonies in each group was counted and compared.

All procedures were performed in accordance with the guidelines for the care and handling of animals prepared by the Iranian Ministry of Health and Medical Education and in line with international conventions on animal experimentation. This study was approved by the Ethics Committee of Kerman University of Medical Sciences, Kerman, Iran (Document No. IR.KMU.REC.1395.200).

**Histopathological assessment**

After euthanizing and sacrificing the animals, tissue specimens were withdrawn from the digestive system, including the stomach and intestine, and transferred into 10% buffered formalin for further fixation. The samples underwent routine tissue processing while a 5- 𝜇m-thick tissue section was prepared and stained. Hematoxylin and eosin (H&amp;E), periodic acid-Schiff, and Gomori methenamine silver were used for staining. The samples were visualized under a light microscope (Olympus BX61) equipped with DP-73 camera. The presence or absence of fungal elements and any inflammatory process were also assessed.

**Statistical analysis**

The statistical analyses between different groups were performed using the independent t-test. The Mann-Whitney U test was used to compare the results of the examined groups based on the sampling time. A p-value less than 0.05 was considered statistically significant. All statistical analyses were performed in SPSS software, version 18.0.

## Results

**Aromatic water essential oil analysis**

The yield percentage of EO extracted from the AW of *T. ammi* was 0.28 mg/mL. The qualitative and quantitative compositions of the EO are presented in Table 1. Approximately, 20 compounds, representing 99.8% area of the EO, were identified. Gas chromatography-mass spectrometry (GC-MS) analyses showed that the main constituent of EO was thymol (78.08%), followed by carvacrol (8.20%) and Mcarvotanacetone (6.50%).

**Table 1:** Chemical composition of essential oil extracted from *Trachyspermum ammi* aromatic water

Compound	KI	Area%
Cyclohexanone (3-methyl)	922.88	0.08
Octanol (3-)	979.84	0.05
Cineole (1,8)	1022.55	0.69
Fenchone	1068.97	0.11
Linalool	1084.09	0.25
Menth-2-en-1-ol (cis-ρ)	1106.87	0.01
Menthone	1133.21	0.04
Isoborneol	1149.10	0.19
Terpinen-4-ol	1162.13	0.94
Terpineol (α-)	1175.18	0.71
Carveol (trans-)	1202.07	0.05
Cumin aldehyde	1214.39	0.20
Carvotanacetone	1221.19	6.50
Anethole (E-)	1259.46	0.06
Thymol	1275.73	78.08
Carvacrol	1282.54	8.20
Piperitenone	1309.6	2.30
Piperitenone oxide	1329.87	0.65
Butylatedhydroxytoluene	1492.24	0.59
Dill apiole	1586.42	0.10
Unknown	-	0.19

KI: Kovats retention index

**Antifungal activities of aromatic water of *Trachyspermum ammi***

The antifungal activities of AW against the standard and clinical strains of *Candida* are shown in Table 2. The growth of the tested yeasts, including azole-resistant strains, was inhibited by the AW of *T. ammi* at a concentration of 0.125-0.25 V/V. Moreover, the AW of *T. ammi* exhibited fungicidal activity against the examined yeasts with the MFC value of 0.25-0.5 V/V.

**Table 2:** Antifungal effect of *Trachyspermum ammi* aromatic water against *Candida* species

Standard *Candida* species		*Trachyspermum ammi*		Fluconazole
Strain	Collection	MIC (V/V)	MFC (V/V)	MIC (µgr/ml)
*C.albicans*	ATCC10261	0.25	0.25	16
*C.albicans*	CBS 562	0.25	0.5	0.25
*C.albicans*	CBS 5982	0.25	0.5	0.25
*C.albicans*	CBS 2730	0.25	0.5	0.5
*C.albicans*	CBS 1912	0.25	0.5	1.0
**C.dubliniensis**	CBS7987	0.25	0.5	1.0
**C.dubliniensis**	CBS 7988	0.25	0.5	1.0
**C.dubliniensis**	CBS 8501	0.25	0.5	1.0
**C.dubliniensis**	CBS 8500	0.125	0.5	0.25
*C.glabrata*	CBS 2175	0.125	0.5	0.25
*C.glabrata*	ATCC90030	0.125	0.5	0.5
*C.glabrata*	CBS 2192	0.125	0.5	0.25
*C.glabrata*	CBS 6144	0.25	0.5	0.5
*C.krusei*	ATCC6258	0.25	0.5	64
*C.parapsilosis*	ATCC4344	0.25	0.25	0.25
*C.tropicalis*	ATCC750	0.25	0.5	2
*C.albicans*	SUMSCC608	0.25	0.25	0.12
*C.tropicalis*	SUMSCC 3086	0.25	0.5	0.12
*C.parapsilosis*	SUMSCC 537	0.25	0.5	0.12
*C.glabrata*	SUMSCC 2303	0.25	0.5	64
*C.albicans*	SUMSCC 625	0.25	0.5	64
**C.dubliniensis**	SUMSCC 634	0.25	0.25	128

ATCC: American type culture collection, CBS: Centraalbureau voor Schimmelcultures, SUMSCC: Shiraz University of Medical Sciences, MIC: minimum inhibitory concentration, MFC: minimum fungicidal concentration

**Inhibition of biofilm formation by aromatic water of *Trachyspermum ammi***

The inhibitory effect of *T. ammi* AW on the formation of biofilm by *C. albicans* was determined by the XTT method. According to the results, the biofilm formation of *C. albicans* was inhibited by 50% and 90% at a concentration of 0.062 V/V and 0.25 V/V, respectively, and the results are shown in Table 3.

**Table 3:** Inhibitory effect of *Candida* biofilm formation by aromatic water of *Trachyspermum ammi* (XTT)

*Trachyspermum ammi*	*C. albicans* (CBS1905)	
Concentration V/V	Mean optical density	%Viability
0 (Negative Cntl.)	0.054	0
1/2	0.062	0.048
1/4	0.074	12.1
1/8	0.075	12.8
1/16	0.146	56.0
1/32	0.169	70.1
1/64	0.180	76.8
1/128	0.182	78.0
1/256	0.209	94.5
1/512	0.210	95.1
1/1024	0.218	100.0
Positive Cntl.	0.218	100.0

Antioxidant activity of aromatic water of *Trachyspermum ammi*

The antioxidant activities of the AW of *T. ammi* and its extracted EO were evaluated by DPPH assay. In this method, DPPH is neutralized by receiving an electron or a hydrogen atom from an antioxidant compound. Based on the results, the EO extracted from *T. ammi* AW showed a considerable antioxidant activity (IC50=69.8±0.8 µg/ml). Similarly, the AW of *T. ammi* revealed a slight antioxidant activity and neutralized DPPH up to 17% (16.92±2.65) at a concentration of 200 mg. Quercetin was used as a standard antioxidant control with an IC50 value of 9.1±0.42 µg/ml.

**Animal modeling**

The numbers of colony-forming units (CFUs) in the mice treated with AW and the controls are shown in Table 4. The mean CFUs obtained from the cultured samples of control and AW groups were 543.5±192.10 and 41.4±18.93, respectively. As expected, no fungal growth was found in the culture of fluconazole-treated mice. The results of the independent sample t-test showed a statistically significant difference between the control and AW groups (*P*=0.02). By splitting the groups based on the sampling time, a significant difference was noted between the control and AW groups in the 5th week. The results are listed in Table 4. These data indicated that the mean CFUs were significantly lower in the AW group in comparison to that in the control group.

**Table 4:** Colony forming units of mice treated with aromatic water and controls based on the sampling time

	Mean±SD		P-value*
	Control	Aromatic water	
Week 3	116.25±96.89	67.75±91.94	0.37
Week 4	250±18.27	28.66±27.59	0.12
Week 5	1406±47.08	19±17.69	0.04

* Mann-Whitney U test

**Figure 1:**
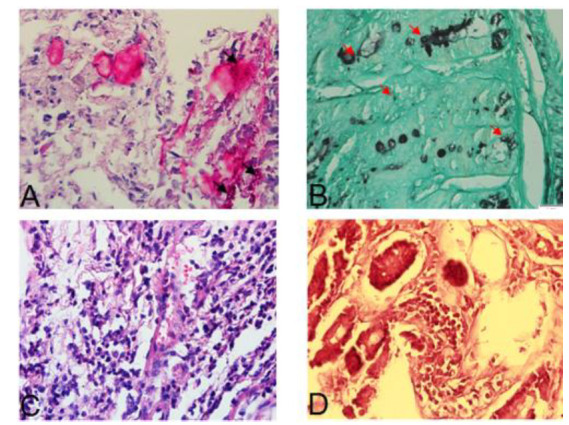
A) *Candida albicans* colonization in glandular part of the stomach associated with necrosis and infiltration of mononuclear inflammatory cells in the control group (PAS, 40x), B) *C. albicans* colonization in the intestinal tissue demonstrating tissue necrosis in the control group (GMS, 20x), C) *C. albicans* elimination in glandular part of the gastric tissue together with absence of yeasts in the group treated with fluconazol (H&E, 40x), D) *C. albicans* elimination in glandular part of the stomach showing a decrease in inflammation and onset of tissue repair in the group treated with *T. ammi* AW (H&E, 20x)

In the histopathological evaluation, the control group revealed necrosis, inflammatory reaction, and yeast colonization in the affected tissue (figures 1A and 1B). Figure 1 presents the removal of *C. albicans* in the tissue after treatment with fluconazole and also a decrease in inflammatory cells. These effects were identical when *T. ammi* AW was administered, suggesting the anti-inflammatory property of *T. ammi* AW (Figure 1D). There was also a healing effect when AW was used, which differentiated AW from fluconazole (Figure 1D).

## Discussion

Since the past centuries, sweet drinking AW has been very popular in many parts of Iran and Middle Eastern countries as a non-alcoholic drink because of their pleasant taste and medicinal properties. The pleasant taste and odor of AW were mainly due to the presence of EO even at low amounts. In other words, the essence is regarded as the active compound of AW.

The chemical compositions of *T. ammi* EOs from different studies are shown in Table 5. Based on the results, the majority of the studies identified thymol as the main compound of *T. ammi* EO at the concentration of 36.4-72.3%. According to the GC-MS analysis, the concentration of thymol (78.08%) in the EO extracted from AW was higher than those of the previous reports [ 18 , 31 - 34 ] and almost identical to the value reported by Kazemi Oskuee et al. [ 35 ].

**Table 5:** Comparison of major chemical compounds of *Trachyspermum ammi* AW essential oil with other studies

Compound	Area %						
	Ref [12]	Ref [25]	Ref [24]	Ref [26]	Ref [27]	Ref [23]	This study
Carvotanacetone-	-	-	-	-	-	6.49
Thymol	36.7	41.8	-	50.07	36.4	72.3	78.08
Carvacrol	0.1	0.6	0.13	0.74	-	8.20
Piperitenone	-	-	-	-	-	-	2.30
p-cymene	21.1	24.4	38	22.90	31.4	-	-
Terpinene(gama)	36.5	27.8	21.07	23.92	21.73	-	-
Pinene (beta)	2.5	1.3	1.42	0.42	1.5	1	-
Terpineolene	-	-	-	-	-	13.2	-
o-cymene	-	-	37.44	0.04	-	11.97	-

Meanwhile, Hassan et al. [ 32 ] did not find thymol in the EO of *T. ammi* and identified p-cymene (38%) as the major constituent of EO. After thymol, phenolic monoterpenes, including carvacrol and carvotanacetone, were found as the most frequent compounds of the EO of AW, while others reported a relatively low amount for these two compounds (up to 0.74%) in EO. Conversely, cymene and γ-terpinene were two dominant compounds of EOs in the majority of previous reports [ 32 ], which were not found in our study. The differences in the chemical composition obtained in our study and those reported by others might be due to the differences in the climate, geographical location, or developmental stage of the cultivated plant.

Furthermore, it has been shown that the chemical compositions of AW and EO are different both qualitatively and quantitatively [ 36 , 37 ]. The difference between the EO composition of AW in our study and those of some previous reports may be due to variation in the boiling points of these compounds. As shown in Table 5, γ- terpinene and p-cymene, as major compounds reported in most studies, are not detected in this study. Such a discrepancy might be due to the low boiling point of these two compounds (173℃ and 177℃, respectively) in comparison to those of thymol and carvacrol (232℃ and 237℃, respectively), and that they might be evaporated much earlier during the hydrodistillation and extraction processing. 

We have previously reported considerable antifungal and antibacterial activities for *T. ammi* EO in vitro [ 18 , 38 ]. In this study, the AW exhibited fungistatic and fungicidal activities against all tested *Candida* species at concentrations ranging from 0.125 V/V to 0.5 V/V. The higher MICs and MFCs of the AW in this study, compared to those of EOs, may be due to the very low amount of EO in AW [ 38 , 39 ]. However, if the dilution factor (yield) of the EO in AW is included (i.e.,0.028%), the inhibitory effect would be comparable or even much better than those of previous reports [ 39 ]. Since the AW of *T. ammi* exhibited inhibitory and cidal activities against fluconazole-resistant *Candida* strains, it can be concluded that the mechanism of action of *T. ammi* AW is different from that of fluconazole.

Moreover, we have shown that the biofilm formation of *Candida* species was inhibited by the EO of *Mentha piperita* at a concentration of up to 2 μl/ml [ 40 ]. In this study, AW inhibited the formation of *Candida* biofilm by about 50% at a concentration of 0.062 V/V. Concerning the very low concentrations of active compounds (i.e., %EO) in the AW, we can conclude that the AW has a high potential to inhibit the formation of biofilm.

The EO of *T. ammi* AW was found to be rich in phenolic monoterpenes. These monoterpenes exhibit their antibacterial activities through the perturbation of the cytoplasmic membrane, resulting in the alteration of membrane permeability and leakage of ions and intracellular materials [ 41 - 43 ]. It has also been reported that thymol and carvacrol have fungicidal activity through the inhibition of ergosterol biosynthesis and the disruption of membrane integrity [ 43 ].

The culture of the stomach of the infected mice and controls indicated that gastric Candidal colonization significantly decreased or even eliminated (in one case) in those receiving the AW of *T. ammi* (for 5 weeks), and this AW had a therapeutic effect on Candidal colonization. Our findings are well correlated with the results of histopathological analyses. The results showed that Candidal colonization declined in the mice following the administration of *T. ammi* AW in therapeutic trials.

On the other hand, reduced inflammation and hyperemia in addition to the start of the healing process in the necrotic tissue were also found in the histopathological examination of the mice that received AW. This phenomenon might be related to the antioxidant activity of the AW or anti-inflammatory activity of thymol and carvacrol as the main constituents of the EO of AW [ 44 , 45 ].

The considerable antifungal activity of AW observed by the elimination of *C. albicans* in the affected tissue might be related to the presence of phenolic monoterpenes in the EO of AW. The antimicrobial and antioxidant activities of AW resulted in considerable effects by reducing inflammation and starting the healing process in the necrotic tissue. These findings can be added to the literature on the treatment of *C. albicans* infection when herbals can be a therapeutic choice to enhance common treatments.

## Conclusion

It seems that the presence of phenolic monoterpenes in AW has resulted in antifungal effects. Pleasant odor and antioxidant properties are extra bonuses to the antimicrobial effects of this plant. Based on the findings, AW might have the potential to be used in the management of alimentary candidiasis or oral hygienic products.

## References

[1] Pfaller MA ( 1996). Nosocomial candidiasis: emerging species, reservoirs, and modes of transmission. Clin Infect Dis.

[2] Kullberg BJ, Arendrup MC ( 2015). Invasive candidiasis. N Engl J Med.

[3] Tobudic S, Kratzer C, Lassnigg A, Presterl E ( 2012). Antifungal susceptibility of Candida albicans in biofilms. Mycoses.

[4] Koh AY, Köhler JR, Coggshall KT, Van Rooijen N, Pier GB ( 2008). Mucosal damage and neutropenia are required for Candida albicans dissemination. PLoS Pathog.

[5] Sonoyama K, Miki A, Sugita R, Goto H, Nakata M, Yamaguchi N ( 2011). Gut colonization by Candida albicans aggravates inflammation in the gut and extra-gut tissues in mice. Med Mycol.

[6] Miranda L, Van der Heijden I, Costa S, Sousa A, Sienra R, Gobara S, et al ( 2009). Candida colonisation as a source for candidaemia. J Hosp Infect.

[7] Morrison G, Collins JS Gastrointestinal infections. Gastrointestinal emergencies. 3rd ed. New Jersey: John Wiley & Sons, Inc.; 2016. P. 251-62.

[8] Whaley SG, Berkow EL, Rybak JM, Nishimoto AT, Barker KS, Rogers PD ( 2017). Azole antifungal resistance in Candida albicans and emerging non-albicans Candida species. Front Microbiol.

[9] Zomorodian K, Moein MR, Rahimi MJ, Pakshir K, Ghasemi Y, Sharbatfar S ( 2011). Possible application and chemical compositions of Carum copticum essential oils against food borne and nosocomial pathogens. Middle East J Sci Res.

[10] Ayatollahi Mousavi SA, Kazemi A ( 2015). In vitro and in vivo antidermatophytic activities of some Iranian medicinal plants. Med Mycol.

[11] Douglas M, Heyes J, Smallfield B Herbs, spices and essential oils: post-harvest operations in developing countries. New York: United Nations Industrial Development Organization/Food and Agriculture Organization of the United Nations (UNIDO/FAO); 2005. P. 61.

[12] Zargari A Medicinal plants. Tehran: Tehran University of Medical Sciences; 1995.

[13] Avicenna A Law in medicine. Tehran: Soroosh; 1988. P. 244-51.

[14] Nickavar B, Abolhasani FA ( 2009). Screening of antioxidant properties of seven Umbelliferae fruits from Iran. Pak J Pharm Sci.

[15] Hejazian SH, Morowatisharifabad M, Mahdavi SM ( 2007). Relaxant effect of Carum copticum on intestinal motility in ileum of rat. World J Zool.

[16] Boskabady M, Shaikhi J ( 2000). Inhibitory effect of Carum copticum on histamine (H1) receptors of isolated guinea-pig tracheal chains. J Ethnopharmacol.

[17] Hejazian SH ( 2006). Analgesic effect of essential oil (EO) from Carum copticum in mice. World J Med Sci.

[18] Goudarzi GR, Saharkhiz M, Sattari M, Zomorodian K ( 2010). Antibacterial activity and chemical composition of Ajowan (Carum copticum Benth. & Hook) essential oil. J Agric Sci Technol.

[19] Rani P, Khullar N ( 2004). Antimicrobial evaluation of some medicinal plants for their anti‐enteric potential against multi‐drug resistant Salmonella typhi. Phytother Res.

[20] Nariman F, Eftekhar F, Habibi Z, Massarrat S, Malekzadeh R ( 2009). Antibacterial activity of twenty Iranian plant extracts against clinical isolates of Helicobacter pylori. Iran J Basic Med Sci.

[21] Satari M, Natanzian GM, Yadgari M, Goudarzi GR, Saharkhiz M ( 2008). Antifungal activity of essential oil and alcoholic extract of Carum copticum against fluconazole resistant and susceptible Candida albicans isolated. Modares J Med Sci.

[22] Bansod S, Rai M ( 2008). Antifungal activity of essential oils from Indian medicinal plants against human pathogenic Aspergillus fumigatus and A. niger. World J Med Sci.

[23] Rasooli I, Fakoor MH, Yadegarinia D, Gachkar L, Allameh A, Rezaei MB ( 2008). Antimycotoxigenic characteristics of Rosmarinus officinalis and Trachyspermum copticum L.essential oils. Int J Food Microbiol.

[24] Raj RK ( 1975). Screening of indigenous plants for anthelmintic action against human Ascaris lumbricoides: Part--II. Indian J Physiol Pharmacol.

[25] Lateef M, Iqbal Z, Rauf U, Jabbar A ( 2006). Anthelmintic activity of Carum copticum seeds against gastro-intestinal nematodes of sheep. J Anim Plant Sci.

[26] Clinical and Laboratory Standards Institute Reference method for broth dilution antifungal susceptibility testing of yeasts; approved standard M27-A3. New York: Clinical and Laboratory Standards Institute; 2008. P. 6-12.

[27] Ramage G, Vandewalle K, Wickes BL, Lopez-Ribot JL ( 2001). Characteristics of biofilm formation by Candida albicans. Rev Iberoam Micol.

[28] Akbari A, Nasiri K, Heydari M, Mosavat SH, Iraji A ( 2017). The protective effect of hydroalcoholic extract of Zingiber officinale Roscoe (Ginger) on ethanol-induced reproductive toxicity in male rats. J Evid Based Complementary Altern Med.

[29] Brand-Williams W, Cuvelier ME, Berset CL ( 1995). Use of a free radical method to evaluate antioxidant activity. LWT-Food Sci Technol.

[30] Conti HR, Huppler AR, Whibley N, Gaffen SL ( 2014). Animal models for candidiasis. Curr Protoc Immunol.

[31] Kim E, Oh CS ( 2016). Antifungal activities after vaporization of ajowan (Trachyspermum ammi) and allspice (Pimenta dioica) essential oils and blends of their constituents against three Aspergillus species. J EssEntial Oil rEsEarch.

[32] Hassan W, Gul S, Rehman S, Noreen H, Shah Z, Mohammadzai I, et al ( 2016). Chemical Composition, essential oil characterization and antimicrobial activity of Carum copticum. Vitam Miner.

[33] Moazeni M, Saharkhiz MJ, Hosseini AA ( 2012). In vitro lethal effect of ajowan (Trachyspermum ammi L. ) essential oil on hydatid cyst protoscoleces. Vet Parasitol.

[34] Mahmoudzadeh M, Hosseini H, Shahraz F, Akhondzadeh‐Basti A, Khaneghah AM, Azizkhani M, et al ( 2017). Essential oil composition and antioxidant capacity of Carum copticum and its antibacterial effect on Staphylococcus aureus, Enterococcus faecalis and Escherichia coli O157: H7. J Food Proc Preserv.

[35] Oskuee RK, Behravan J, Ramezani M ( 2011). Chemical composition, antimicrobial activity and antiviral activity of essential oil of Carum copticum from Iran. Avicenna J Phytomed.

[36] Ciccarelli D, Noccioli C, Pistelli L ( 2013). Chemical composition of essential oils and aromatic waters from different Italian Anthemis maritima populations. Chem Biodiversity.

[37] Gallori S, Flamini G, Bilia AR, Morelli I, Landini A, Vincieri FF ( 2001). Chemical composition of some traditional herbal drug preparations: Essential oil and aromatic water of costmary (Balsamita suaveolens Pers.). J Agri Food Chem.

[38] Zomorodian K, Ghadiri P, Saharkhiz MJ, Moein MR, Mehriar P, Bahrani F, et al ( 2015). Antimicrobial activity of seven essential oils from Iranian aromatic plants against common causes of oral infections. Jundishapur J Microbiol.

[39] Ashrafi Tamai I, Zahraei Salehi T, Khosravi AR, Sharifzadeh A, Balal A ( 2013). Chemical composition and anti-candida activity of Trachyspermum ammi essential oil on azoles resistant Candida albicans isolates from oral cavity of HIV+ patients. J Med Plants.

[40] Saharkhiz MJ, Motamedi M, Zomorodian K, Pakshir K, Miri R, Hemyari K ( 2012). Chemical composition, antifungal and antibiofilm activities of the essential oil of Mentha piperita L. ISRN Pharm.

[41] Guo N, Liu J, Wu X, Bi X, Meng R, Wang X, et al ( 2009). Antifungal activity of thymol against clinical isolates of fluconazole- sensitive and-resistant Candida albicans. J Med Microbiol.

[42] Chami N, Bennis S, Chami F, Aboussekhra A, Remmal A ( 2005). Study of anticandidal activity of carvacrol and eugenol in vitro and in vivo. Oral Microbiol Immunol.

[43] Ahmad A, Khan A, Akhtar F, Yousuf S, Xess I, Khan L, et al ( 2011). Fungicidal activity of thymol and carvacrol by disrupting ergosterol biosynthesis and membrane integrity against Candida. Eur J Clin Microbiol Infect Dis.

[44] Braga PC, Dal Sasso M, Culici M, Bianchi T, Bordoni L, Marabini L ( 2006). Anti-inflammatory activity of thymol: inhibitory effect on the release of human neutrophil elastase. Pharmacology.

[45] Boskabady MH, Alitaneh S, Alavinezhad A ( 2014). Carum copticum L.: a herbal medicine with various pharmacological effects. Biomed Res Int.

